# In vitro and in vivo physiology of low nanomolar concentrations of Zn^2+^ in artificial cerebrospinal fluid

**DOI:** 10.1038/srep42897

**Published:** 2017-02-17

**Authors:** Haruna Tamano, Ryusuke Nishio, Yukina Shakushi, Miku Sasaki, Yuta koike, Misa Osawa, Atsushi Takeda

**Affiliations:** 1 Department of Neurophysiology, School of Pharmaceutical Sciences, University of Shizuoka, 52- 1 Yada, Suruga-ku, Shizuoka 422-8526, Japan

## Abstract

Artificial cerebrospinal fluid (ACSF), i.e., brain extracellular medium, which includes Ca^2+^ and Mg^2+^, but not other divalent cations such as Zn^2+^, has been used for *in vitro* and *in vivo* experiments. The present study deals with the physiological significance of extracellular Zn^2+^ in ACSF. Spontaneous presynaptic activity is suppressed in the stratum lucidum of brain slices from young rats bathed in ACSF containing 10 nM ZnCl_2_, indicating that extracellular Zn^2+^ modifies hippocampal presynaptic activity. To examine the *in vivo* action of 10 nM ZnCl_2_ on long-term potentiation (LTP), the recording region was perfused using a recording electrode attached to a microdialysis probe. The magnitude of LTP was not modified in young rats by perfusion with ACSF containing 10 nM ZnCl_2_, compared to perfusion with ACSF without Zn^2+^, but attenuated by perfusion with ACSF containing 100 nM ZnCl_2_. Interestingly, the magnitude of LTP was not modified in aged rats even by perfusion with ACSF containing 100 nM ZnCl_2_, but enhanced by perfusion with ACSF containing 10 mM CaEDTA, an extracellular Zn^2+^ chelator. The present study indicates that the basal levels of extracellular Zn^2+^, which are in the range of low nanomolar concentrations, are critical for synaptic activity and perhaps increased age-dependently.

Divalent cations such as Ca^2+^ and Mg^2+^ play key roles for synaptic neurotransmission^1^. Among divalent cations, Ca^2+^ concentration is the highest in the brain and is approximately 1.2 mM in the cerebrospinal fluid (CSF) and brain extracellular fluid in the adult rats^2^. The influx of extracellular Ca^2+^ into neurons is essential for strengthening of synaptic connections between neurons, i.e., synaptic plasticity such as long-term potentiation (LTP)^3^,^4^. Approximately 2 mM Ca^2+^ is added to conventional artificial cerebrospinal fluid (ACSF) based on essentiality of intracellular Ca^2+^ signaling in neurons and glial cells, which is induced by the influx of extracellular Ca^2+^. In contrast, excess influx of extracellular Ca^2+^ into neurons leads to the pathophysiological process of neurodegeneration associated with glutamate excitotoxicity^5^,^6^,^7^.

Zinc serves as zinc metalloproteins and is also essential for synaptic neurotransmission^8^. Furthermore, a subclass of glutamatergic neurons is zincergic and is stained by Timm’s sulfide-silver method, which stains histochemically reactive zinc in the presynaptic vesicles. Zn^2+^ is co-released with glutamate in activity-dependent manner^9^ and play a role as a signal factor as well as Ca^2+^ 10. It has been reported that synaptic Zn^2+^ signaling is critical for LTP induction in the hippocampus. At mossy fiber-CA3 pyramidal cell synapses and Schaffer collateral-CA1 pyramidal cell synapses, which are zincergic, extracellular Zn^2+^, which is increased by LTP induction, serves for intracellular Zn^2+^ signaling and is involved in learning and memory^11^,^12^,^13^,^14^. Even at medial perforant pathway-dentate granule cell synapses, which are non-zincergic, intracellular Zn^2+^ signaling, which originates in the internal stores containing Zn^2+^, is involved in memory acquisition and retention^15^,^16^.

Zn^2+^ concentration in the brain extracellular fluid, which is estimated to be approximately 10 nM in the adults^17^, is much lower than Ca^2+^ concentration. Thus, much less attention has been paid to essentiality of Zn^2+^ in brain extracellular fluid for synaptic function. ACSF, i.e., brain extracellular medium, without Zn^2+^ has been used for *in vitro* and *in vivo* experiments. It is possible that not only neuronal excitation but also synaptic plasticity such as LTP is modified in brain slices bathed in ACSF without Zn^2+^, in which original physiology might not appear^18^. Clarifying the action of extracellular Zn^2+^ in the range of physiological concentrations is important to understand synaptic function precisely and also to understand the bidirectional action of Zn^2+^ under physiological and pathological conditions. Low nanomolar concentrations of Zn^2+^ are more physiologically relevant than micromolar concentrations of Zn^2+^ widely used, which are often neurotoxic. To assess the physiological range of extracellular Zn^2+^ concentration, in the present study, the action of low nanomolar concentrations of Zn^2+^ added to ACSF was examined in both *in vitro* and *in vivo* experiments using young and old rats.

## Results

### Spontaneous presynaptic activity

**Figure 1:**
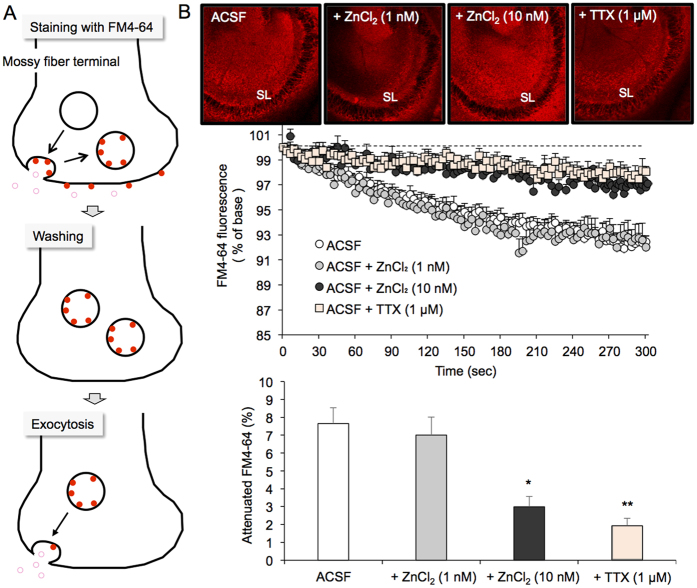
Suppression of spontaneous mossy fiber exocytosis of young rats in the presence of 10 nM ZnCl_2_. (**A**) Schematic illustration of mossy fiber exocytosis assessed by attenuation of FM4-64 fluorescence. (**B**) FM4-64 fluorescence images in the CA3 after loading FM4-64 into slices bathed in ACSF, ACSF containing 1 nM Zn^2+^, and ACSF containing 10 nM Zn^2+^, and FM4-64 fluorescence image in the CA3 after loading of FM4-64 into slices bathed in ACSF and transferring the slices to ACSF containing 1 μM TTX (time 0 sec) (upper). Each point and line (mean ± SEM) represents the changes in FM4-64 fluorescence in the stratum lucidum (SL) of brain slices bathed in ACSF (n = 17), 1 nM ZnCl_2_ (n = 5), 10 nM ZnCl_2_ (n = 8) and 1 μM TTX (n = 6) after loading FM4-64 (time 0), which is expressed as 100%. (n = 6) (middle). Each bar and line (the mean ± SEM) represents the rate of the decreased FM4-64 fluorescence at time 300 sec (lower). *p < 0.05; **p < 0.01, vs. ACSF (Tukey’s test).

Spontaneous presynaptic activity, which was determined by the attenuation of FM4-64 fluorescence, was assessed in the stratum lucidum containing CA3 mossy fiber boutons of slices bathed in agents in ACSF (Fig. 1). FM4-64 fluorescence intensity was almost the same among four groups at the start of observation (time 0 sec) (upper images in Fig. 1B). Presynaptic activity in brain slices bathed in ACSF without Zn^2+^ was significantly higher than that in brain slices bathed in 10 nM ZnCl_2_ in ACSF, which was comparable with presynaptic activity in brain slices bathed in ACSF without Zn^2+^ under inhibition of neuronal depolarization in the presence of TTX (Fig. 1B). Presynaptic activity in brain slices bathed in 1 nM ZnCl_2_ was almost the same as that bathed in ACSF without Zn^2+^.

**Figure 2:**
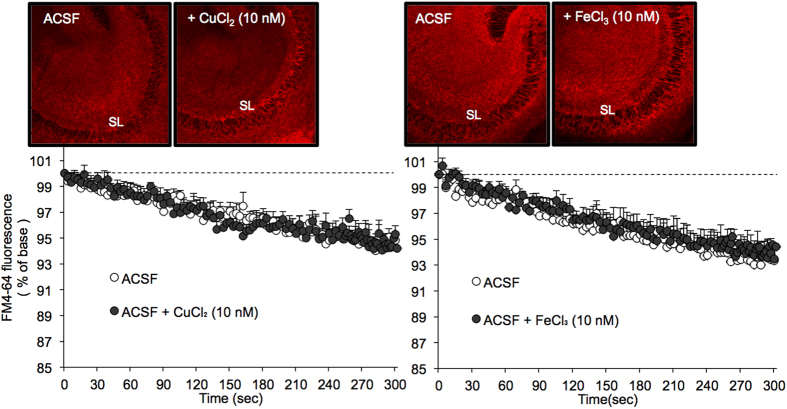
No effect of 10 nM CuCl_2_ and 10 nM FeCl3 on spontaneous mossy fiber exocytosis of young rats. FM4-64 fluorescence images in the CA3 after loading FM4-64 into slices bathed in ACSF and ACSF containing 10 nM Cu^2+^ or ACSF containing 10 nM Fe^3+^ (time 0 sec) (upper). Each point and line (mean ± SEM) represents the changes in FM4-64 fluorescence in the stratum lucidum (SL) of brain slices bathed in ACSF (n = 10), 10 nM CuCl_2_ (n = 6), and 10 nM FeCl_3_ (n = 6) after loading FM4-64 (time 0), which is expressed as 100%. (n = 6) (lower).

FM4-64 fluorescence intensity was almost the same between slices bathed in ACSF and ACSF containing 10 nM CuCl_2_ or ACSF containing 10 nM FeCl_3_ at the start of observation (time 0 sec) (upper images in Fig. 2). Presynaptic activity in brain slices was not modified by bathing in 10 nM CuCl_2_ or 10 nM FeCl_3_ in ACSF (Fig. 2).

### Intracellular levels of Ca^2+^ in the hippocampus

**Figure 3:**
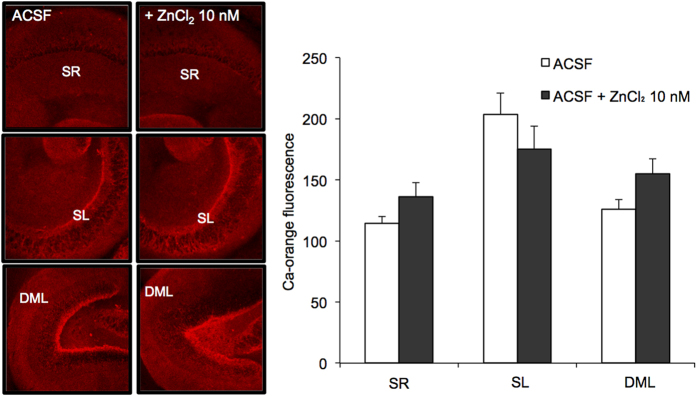
Intracellular Ca^2+^ imaging in the hippocampus of young rats with calcium orange AM. Intracellular calcium orange fluorescence was imaged to estimate the basal levels of cytosolic Ca^2+^ in the hippocampus of brain slices bathed in ACSF (n = 7) and 10 nM ZnCl_2_ in ACSF (n = 7). SR, stratum radiatum; SL, stratum lucidum; DML. dentate molecular layer (left). Each bar and line (the mean ± SEM) represents fluorescent intensity in the SR, SL, and DML.

Variations in the intracellular levels of Ca^2+^ were compared between brain slices prepared with conventional ACSF without Zn^2+^ and bathed in ACSF containing 10 nM ZnCl_2_. Intracellular Ca^2+^ levels determined with calcium orange were also almost the same in the stratum radiatum, stratum lucidum and dentate molecular layer between the two groups (Fig. 3).

### *In vivo* dentate gyrus LTP

**Figure 4:**
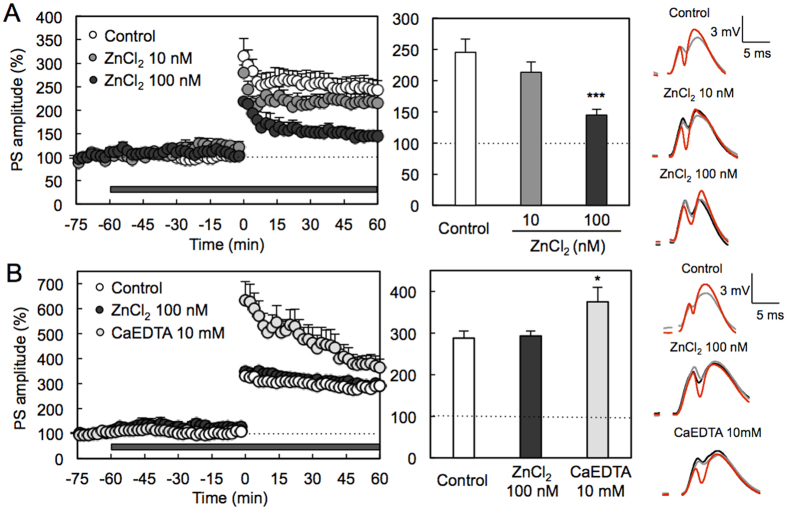
Action of extracellular Zn^2+^ and extracellular Zn2^+^ chelator in dentate gyrus LTP of young and aged rats. (**A**) Recording region was perfused with ACSF in young rats and then 10 nM (n = 5) and 100 nM (n = 4) ZnCl_2_ in ACSF as shown by the shaded bar and HFS (10 trains of 20 pulses at 200 Hz separated by 1 s) was delivered at time 0 min (left). Each bar and line (mean ± SEM) represents the averaged PS amplitude of the last 10 min (time 50–60 min) (middle). ***p < 0.001, vs. control (ACSF) (n = 10) (Tukey’s test). Representative fEPSP recordings at time −70 (black line), −30 (grey line) and 50–60 min (red line) are shown (right). (**B**) Recording region was perfused with ACSF in aged rats and then 100 nM (n = 18) and 10 mM CaEDTA (n = 7) ZnCl_2_ in ACSF as shown by the shaded bar and HFS (10 trains of 20 pulses at 200 Hz separated by 1 s) was delivered at time 0 min (left). Each bar and line (mean ± SEM) represents the averaged PS amplitude of the last 10 min (time 50–60 min) (middle). *p < 0.05, vs. control (ACSF) (n = 26) (Tukey’s test). Representative fEPSP recordings at time −70 (black line), −30 (grey line) and 50–60 min (red line) are shown (right).

To pursue physiological range of extracellular Zn^2+^ concentration, *in vivo* LTP induction was compared between local perfusion of the recording area with ACSF without Zn^2+^ and ACSF containing Zn^2+^. LTP was not attenuated under perfusion with 10 nM ZnCl_2_, but significantly attenuated under perfusion with 100 nM ZnCl_2_ (Fig. 4A).

Adlard *et al*.^19^ report that metal chaperones for zinc and cupper, i.e., clioquinol and PBT2, prevent normal age-related cognitive decline and demonstrate that the metal chaperones are effective for preventing the zinc-mediated cognitive decline that is observed in aging and diseases, suggesting that extracellular Zn^2+^ concentration in the hippocampus is potentially changed along with aging. When LTP was induced under perfusion with 100 nM ZnCl_2_ in aged rats, it was not attenuated unlike the case of young rats (Fig. 4A and B). Interestingly, LTP was significantly enhanced under perfusion with 10 mM CaEDTA, an extracellular Zn^2+^ chelator, in aged rats (Fig. 4B), although no effect of 10 mM CaEDTA on LTP is reported in young rats^15^.

## Discussion

Zn^2+^ -deficient ACSF has a negative impact on *in vitro* brain slice preparation and experiments. Hippocampal excitability is attenuated in brain slices pretreated with ACSF containing 20 nM ZnC1_2_ for 1 h, compared with those pretreated with conventional ACSF without Zn^2+^ 20. An opposite effect on extracellular Zn^2+^ is observed between *in vitro* and *in vivo* LTP induction; *in vitro* CA1 LTP is enhanced in hippocampal slices bathed in 5 μM ZnC1_2_ 21,22, while *in vivo* CA1 LTP is attenuated under local perfusion of the recording region with 0.1–1 μM ZnCl_2_ by using a recording electrode attached to a microdialysis probe^14^. The evidence suggests that original synaptic activity is modified in slice experiments using ACSF without Zn^2+^ and that addition of Zn^2+^ to ACSF is necessary to prevent modification of synaptic function.

To clarify the physiological range of extracellular Zn^2+^ concentration, the action of low nanomolar concentrations of Zn^2+^ in ACSF was examined in both *in vitro* and *in vivo* experiments. On the basis of the estimated concentration of extracellular Zn^2+^ under the basal (static) condition, which is approximately 10 nM^17^, the present study performed focused on the concentration of 10 nM. Spontaneous presynaptic activity assessed with FM4-64 is significantly suppressed in the stratum lucidum of brain slices from young rats bathed in ACSF containing 10 nM Zn^2+^, but not in ACSF containing 10 nM Cu^2+^ or 10 nM Fe^3+^, indicating that hippocampal presynaptic activity is enhanced in brain slices prepared with ACSF without Zn^2+^. Micromolar Zn^2+^ also suppresses hippocampal mossy fiber exocytosis^23^. It is likely that Zn^2+^ dose-dependently suppresses presynaptic activity in the hippocampus. On the other hand, the basal levels of intracellular Ca^2+^ determined with calcium orange were not modified in the hippocampus of brain slices prepared with ACSF without Zn^2+^. Suh *et al*.^24^ report that acute brain slice preparations are poorly suitable for research on roles of endogenous Zn^2+^ released from zincergic neurons. Vesicular zinc determined by Timm’s sulfide-silver method is lost during slice preparation and slice incubation; *in vitro* Zn^2+^ release is reduced to about 25% of *in vivo* Zn^2+^ release. It is likely that extracellular Zn^2+^ is physiologically critical in the range of low nonomolar concentrations for *in vitro* slice experiments from young animals.

To examine the action of 10 nM Zn^2+^ on *in vivo* LTP induction, the recording region was perfused using a recording electrode attached to a microdialysis probe. The magnitude of LTP was not significantly modified in young rats by perfusion with ACSF containing 10 nM Zn^2+^, compared to perfusion with ACSF without Zn^2+^, but attenuated by perfusion with ACSF containing 100 nM Zn^2+^. Because 100 nM Zn^2+^ also attenuates CA1 LTP^14^, this concentration of Zn^2+^ is beyond the range of physiological concentration in young rats as the basal concentration of extracellular Zn^2+^. The present study indicates that extracellular Zn^2+^ modulates LTP at low nanomolar concentrations in young rats. Interestingly, the magnitude of LTP was not modified in aged rats by perfusion with ACSF containing 100 nM Zn^2+^. The perfusate reaches the equilibrium state with the brain extracellular fluid containing Zn^2+^, resulting in the perfusate (ACSF) with Zn^2+^. It is possible that Zn^2+^ concentration in the perfusate during the perfusion is higher in aged rats than in young rats. The magnitude of LTP was enhanced in aged rats by perfusion with ACSF containing 10 mM CaEDTA, while it is not modified in young rats under the same condition^15^. It is likely that extracellular Zn^2+^ concentration is increased age-dependently and that 100 nM Zn^2+^ is in the range of physiological concentration in aged rats. Extracellular Zn^2+^ may suppressively modulate LTP induction, especially in aged rats.

Zinc concentration in the CSF is reported to be 150–380 nM^25^,^26^,^27^. If it is the same as zinc concentration in the brain extracellular fluid, a large portion of zinc is not free ion in the brain extracellular fluid under the basal condition. At zincergic synapses, extracellular Zn^2+^ levels are dynamically changed by the degree of Zn^2+^ activity-dependently released from neuron terminals, which is required for learning and memory via synaptic plasticity^14^. Even at non-zincergic synapses, extracellular Zn^2+^ may serve as a pool for Zn^2+^ release from the internal stores, which is also required for learning and memory^15^. In conclusion, the present study indicates that original neurophysiology may not appear in ACSF without Zn^2+^. The basal levels of extracellular Zn^2+^, which are in the range of low nanomolar concentrations, are critical for synaptic activity and perhaps increased age-dependently. Homeostasis of Zn^2+^ in both extracellular and intracellular compartments is still poorly understood and the understanding is important to search a strategy for preventing Zn^2+^ -mediated cognitive decline.

## Experimental Procedures

### Chemicals and animals

Male Wistar rats were purchased from Japan SLC (Hamamatsu, Japan) and used as young (6–9 weeks of age) and aged (>60 weeks of age) rats. They were housed under the standard laboratory conditions (23 ± 1 °C, 55 ± 5% humidity) and had access to tap water and food ad libitum. FM4–64, an indicator of presynaptic activity, and calcium orange AM, a membrane-permeable calcium indicator, were purchased from Sigma-Aldrich (St. Louis, MO) and Molecular Probes, Inc. (Eugene, OR), respectively. These indicators were dissolved in dimethyl sulfoxide (DMSO) and then diluted to artificial cerebrospinal fluid (ACSF) containing 119 mM NaCl, 2.5 mM KCl, 1.3 mM MgSO_4_, 1.0 mM NaH_2_PO_4_, 2.5 mM CaCl_2_, 26.2 mM NaHCO_3_, and 11 mM D-glucose (pH 7.3). ZnCl_2_, FeCl_3_ and CuCl_2_ were dissolved in purified water and prepared as 10 mM stock solutions. The stock solutions were diluted to 1 μM with purified water. One micromolar metals in water were diluted with ACSF and nanomolar solutions were freshly prepared.

### Brain slice preparation

Mice were anesthetized with ether and decapitated. The brain was quickly removed and immersed in ice-cold choline-ACSF containing 124 mM choline chloride, 2.5 mM KCl, 2.5 mM MgCl_2_, 1.25 mM NaH_2_PO_4_, 0.5 mM CaCl_2_, 26 mM NaHCO_3_, and 10 mM glucose (pH 7.3) to suppress excessive neuronal excitation. Horizontal brain slices (400 μm) were prepared by using a vibratome ZERO-1 (Dosaka Kyoto, Japan) in an ice-cold choline-ACSF. Slices were then maintained in ACSF and ACSF containing 1–10 nM ZnCl_2_, 10 nM CuCl_2_, or 10 nM FeCl_3_ at 25 °C for 1 h. All solutions used in the experiments were continuously bubbled with 95% O_2_ and 5% CO_2_.

### Spontaneous exocytosis

The brain slices bathed in ACSF and ACSF containing Zn^2+^, Cu^2+^, or Fe^3+^ were transferred to an incubation chamber filled with each ACSF containing 5 μM FM4-64, 45 mM KCl, and 10 μM 6-cyano-7-nitroquinoxaline-2,3-dione (CNQX), an antagonist of AMPA/kainate receptors, allowed to stand at 25 °C for 90 s and transferred a chamber filled with each ACSF to wash out extracellular FM4-64 and KCl for 15 min. Brain slices were transferred to a recording chamber filled with each ACSF containing 10 μM CNQX to prevent recurrent activity. FM4-64 fluorescence (excitation, 543 nm; emission, 640 nm) was measured with a confocal laser-scanning microscopic system LSM 510 META at the rate of 1 Hz for 300 s through a 10 × objective. In another experiment, the brain slices bathed in ACSF was treated in the same manner for staining with FM4-64 and transferred to a recording chamber filled with 1 μM tetrodotoxin (TTX), a voltage-gated sodium channel blocker, in ACSF containing 10 μM CNQX to measure the change in FM4-64 fluorescence under inhibition of neuronal depolarization. Because FM4-64 fluorescence originates in vesicular membrane-bound FM4-64, FM4-64 fluorescence is attenuated by spontaneous presynaptic activity^28^,^29^ (Fig. 1A). FM4-64 fluorescence was then normalized by the initial fluorescence intensity at the time 0 sec, which is expressed as 100%. The rate (%) of attenuated FM4-64 fluorescence at the time 300 sec was compared among groups bathed in ACSF and reagents in ACSF.

### Intracellular levels of Ca^2+^

To assess the basal levels of intracellular Ca^2+^, the brain slices in ACSF and ACSF containing 10 nM ZnCl_2_ were placed for 30 min in each ACSF containing 5 μM calcium orange AM, transferred to a chamber filled with each ACSF to wash out extracellular calcium orange AM for 15 min, and transferred to a recording chamber filled with each ACSF. The fluorescence of calcium orange (excitation, 543 nm; monitoring, above 560 nm) was measured with a confocal laser-scanning microscopic system LSM 510 META for 30 sec at the rate of 1 Hz through a 10 × objective.

### *In vivo* LTP

Male rats were anesthetized with chloral hydrate (400 mg/kg) and placed in a stereotaxic apparatus. A bipolar stimulating electrode and a monopolar recording electrode made of tungsten wire attached to an microdialysis probe (E-A-I-12-01, Eicom Co., Kyoto) were positioned stereotaxically so as to selectively stimulate the perforant pathway while recording in the dentate gyrus under local perfusion with agents in ACSF (127 mM NaCl, 2.5 mM KCl, 0.9 mM MgCl_2_, 1.2 mM NaH_2_PO_4_, 1.3 mM CaCl_2_, 21 mM NaHCO_3_, and 3.4 mM D-glucose (pH 7.3)). The electrode stimulating the perforant pathway was positioned 8.0 mm posterior to the bregma, 4.5 mm lateral, 3.0–3.5 mm inferior to the dura. A recording electrode was implanted ipsilaterally 4.0 mm posterior to the bregma, 2.3–2.5 mm lateral and 3.0–3.5 mm inferior to the dura. All the stimuli were biphasic square wave pulses (200 μs width) and their intensities were set at the current that evoked 40% of the maximum population spike (PS) amplitude. Test stimuli (0.05 Hz) were delivered at 20 s intervals to monitor PS amplitude. At the beginning of the experiments, input/output curves were generated by systematic variation of the stimulus current (0.1–5.0 mA) to evaluate synaptic potency. After stable baseline recording under perfusion with ACSF for at least 30 min, PS amplitudes were measured under perfusion with agents in ACSF for 60 min and LTP was induced by delivery of high-frequency stimulation (HFS; 10 trains of 20 pulses at 200 Hz separated by 1 s) and recorded for 60 min. PS amplitudes (test frequency: 0.05 Hz) were averaged over 120-second intervals and expressed as percentages of the mean PS amplitude measured during the 15-min baseline period (from −75 min ~ to −60 min) perfused with ACSF prior to LTP induction, which was expressed as 100%. PS amplitudes for the last 10 min were also averaged and represented as the magnitude of LTP.

### Statistical analysis

For statistical analysis, Student’s *t*-test was used for comparison of the means of unpaired or paired two-data. For multiple comparisons, differences between the control and treatments were assessed by one-way ANOVA followed by post hoc testing using the Tukey’s test (the statistical software, GraphPad Prism 5). A value of p < 0.05 was considered significant. Data were expressed as means ± standard error. The results of statistical analysis are described in each figure legend. In Figs 1, 2 and 3, “n” means the number of slices. The experiments have separately done in triplicate to confirm the reproducibility and all slices used in the present study were analyzed statistically. In Fig. 4, “n” means the number of rats.

### Ethics Statement

All experiments were performed in accordance with the Guidelines for the Care and Use of Laboratory Animals of the University of Shizuoka that refer to American Association for Laboratory Animals Science and the guidelines laid down by the NIH (NIH Guide for the Care and Use of Laboratory Animals) in the USA. The ethics committee of the University of Shizuoka has approved all experimental protocols (The approval number, 136043).

## Additional Information

**How to cite this article**: Tamano, H. *et al*. In vitro and in vivo physiology of low nanomolar concentrations of Zn^2+^ in artificial cerebrospinal fluid. *Sci. Rep.* **7**, 42897; doi: 10.1038/srep42897 (2017).

**Publisher's note:** Springer Nature remains neutral with regard to jurisdictional claims in published maps and institutional affiliations.
